# Exhaustive Description of the System Architecture and Prototype Implementation of an IoT-Based eHealth Biometric Monitoring System for Elders in Independent Living

**DOI:** 10.3390/s21051837

**Published:** 2021-03-06

**Authors:** Cristian Vizitiu, Călin Bîră, Adrian Dinculescu, Alexandru Nistorescu, Mihaela Marin

**Affiliations:** 1 Space Applications for Human Health and Safety Department, Institute of Space Science, 077125 Măgurele, Romania; calin.bira@upb.ro (C.B.); alexnistorescu@spacescience.ro (A.N.); mihaela13@spacescience.ro (M.M.); 2 Devices, Circuits, Electronic Architectures, Faculty of Electronics, Telecommunications and Information Technology, University Politehnica of Bucharest, 060042 Bucharest, Romania; 3 Image Processing and Analysis Laboratory, Faculty of Electronics, Telecommunications and Information Technology, University Politehnica of Bucharest, 060042 Bucharest, Romania; 4 Faculty of Science Physical Education and Informatics, University of Pitesti, 110040 Pitesti, Romania

**Keywords:** e-Health, internet of things (IoT), elders, independent living, active and assisted living (AAL), systems engineering, biometric sensors, arduino, noncommunicable diseases (NCDs), choice reaction time (CRT)

## Abstract

In this paper, we present an exhaustive description of an extensible e-Health Internet-connected embedded system, which allows the measurement of three biometric parameters: pulse rate, oxygen saturation and temperature, via several wired and wireless sensors residing to the realm of Noncommunicable Diseases (NCDs) and cognitive assessment through Choice Reaction Time (CRT) analysis. The hardware used is based on ATMEGA AVR + MySignals Hardware printed circuit board (Hardware PCB), but with multiple upgrades (including porting from ATMEGA328P to ATMEGA2560). Multiple software improvements were made (by writing high-level device drivers, text-mode and graphic-mode display driver) for increasing functionality, portability, speed, and latency. A top-level embedded application was developed and benchmarked. A custom wireless AT command firmware was developed, based on ESP8266 firmware to allow AP-mode configuration and single-command JavaScript Object Notation (JSON) data-packet pushing towards the cloud platform. All software is available in a git repository, including the measurement results. The proposed eHealth system provides with specific NCDs and cognitive views fostering the potential to exploit correlations between physiological and cognitive data and to generate predictive analysis in the field of eldercare.

## 1. Introduction

A special focus in worldwide healthcare perspective is oriented on chronic diseases, also known as Noncommunicable Diseases (NCDs), given by numerous risk factors especially in terms of poor income, environmentally and metabolic stressors, physical inactivity, and negative behavior patterns, which lead to 71% of all deaths globally, whereas over 85% of these deaths, considered as prematurely, emerge in low- and middle- income from underdeveloped countries. According to World Health Organization (WHO), ageing population represents a significantly affected segment by the NCDs [1], especially in the case of increased rate of the elder segment as forecasted in the next decades, where several public health recommendations shall be considered in terms of assistive technologies implementations, caregiver support, home concierge services, specific policies, and regulations for protecting elders [2].

Given the fast increase of elder population in the global population (by 120% until 2050), the impact of NCDs, plus neurological illnesses [3], and in addition considering the current pandemic situation, Internet of Things (IoT) technologies are the best-case scenario in detecting and alerting caregivers or emergency services in case of changes that can become a threat to the patient life.

Fortunately, to date, e-Health systems have proven to have great potential in providing telediagnosis, teleconsultancy, telemonitoring processes for elders and specialists within the same or different locations with the main benefits of providing access for elders to supportive care from specialized stakeholders (e.g., caregivers, volunteers, physicians etc.) and at the same time lowering the elderly caregiving and healthcare costs [4].

Nevertheless, as an in-depth literature review published in 2020 clearly reveals [5] that there are significant challenges of the IoT technologies acceptance in terms of user interfaces, easy-to-use features, size, weight, and obtrusiveness, while the measurement accuracy, especially in the realm of cognition, was addressed in just four studies as reported. The cognition field refers to Mild Cognitive Impairments (MCIs), dementia, and severe forms of it, namely Alzheimer’s Disease (AD), a neurodegenerative disorder.

The connection between NCDs and cognitive impairments for the elderly has been more evidenced in the literature [6,7], with MCI emergence in elders with cardiovascular disease (i.e., 35%) [8], pulmonary disease (i.e., 25%) [8], and diabetes mellitus disease (i.e., 20%) [9] being highlighted. As aging influences the decline of cognitive components, like alertness, attention, reduced stimulus perception, and decision making, there were several experiments taken into consideration, especially in the last decades, regarding Choice Reaction Time (CRT) and Simple Reaction Time (SRT) approaches [10] for consolidating conclusions in terms of increased reaction time to visual stimuli with age [11].

## 2. Related Work

As “Internet of Things” semantically means “a world-wide network of interconnected objects uniquely addressable based on standard communication protocols” [12], in the last few years, the IoT market has seen a continuous technological increase and aims to connect a wide range of devices and systems from sensors and actuators, appliances, computers, and cellular phones. The main challenge envisaged is to create a complex system that can be integrated “from the supply chain to bedside” but also to be included in the everyday life of elders to track physiological and psychological changes before the need of hospital care occurs. Considering the demographic trends that show an ascending need of assistance for elders, it is desirable to create a system capable of communicating with other devices and human beings in order for elderly to live longer and more safely at home [12,13].

Asghari, P. et al. [14] defines IoT as an ecosystem that includes smart devices with sensors, integrated into a network in order to provide to the end users a smart services environment. Some relevant case studies of examples of IoT wearable sensors and devices for eldercare have been identified in the literature as follows.

Bose et al. [15] developed a sensor that measures body temperature, heartbeat, acceleration, and blood pressure; the collected data are transferred to the remote-control station via a developed wireless mesh network architecture (consists of Tags with sensors, Routers and Coordinator/Gateway). They used an IEEE 802.15.4/Zigbee protocol for WPAN (Wireless Personal Area Network) in order to provide a low-cost, low-power wireless mesh network and longer battery life.

Aljehani et al. [16] developed an application for Apple smartwatch that measures heart rate and locates the patient based on the Internet of Things (IoT) concept. The solution has two parts: (i) The Alzheimer’s patient wears an IoT device, which is the Apple Smartwatch, and (ii) an iOS application on the caregiver’s iPhone connected to the patient’s watch in order to access and store the patient’s heart rate and location data.

Karakaya et al. [17] used a smart watch (with an accelerometer and gyroscope) as an IoT with a mobile app in order to identify daily activities such as walking, sitting, and falling with the purpose of predicting elderly people’s activities.

Srinivas et al. [18] developed a health monitoring system based on cloud user authentication. The main objective of their work was to ensure a secure user authentication scheme in order to access real-time data acquired by the wearable sensors deployed on the patient’s body. 

Farman et al. [19] elaborated a diagnosis engine system able to predict heart disease with an 98.5% accuracy and recommend dietary plans and activities. Their proposed solution has two main data sources: The first one is the wireless body sensor network based on medical sensors that acquires internal and external physiological data (electrocardiogram, electroencephalogram, heart rate, blood pressure, position, activities, respiration rate, blood sugar, oxygen saturation, and cholesterol levels), transmitted via Bluetooth and Wi-Fi devices, and the second data source is Electronic Medical Records.

In the realm of cognitive assessment and assistive technologies, there are several solutions developed for diagnosing AD by gait postural control analysis [20], stimulating cognitive functions via SenseCam diary method of remembering images [21], MCI and dementia diagnosis through number-letter cognitive tests deployed on PC correlated with wearable inertial sensors [22], and preserving cognitive functions through the eWall Open-Source Cloud-Based eHealth Platform able to provide cognitive training platforms under the shape of video games [23].

Thus, IoT applications based on embedded devices endowed with computational and Internet communication capabilities present a new paradigm and huge potential for modern healthcare and eldercare. [24],

Common solutions in monitoring health parameters of patients in wards are based on commercial off the shelf (COTS) wearable devices collecting different biometric data via Bluetooth and advancing it to Internet via a Gateway, most often in the form of smartphones, solutions coming with disadvantages in terms of high components price and increased power consumption, implicitly in less autonomy, enhanced communication delay, and high overall cost of the eHealth system. In contrast, using ESP8266 as Wi-Fi node integrated in the IoT sensors may be an avenue towards low-cost (e.g., prices between $4.00 to $10.00) and reduced power consumption solutions (i.e., being endowed with three configurable sleep modes) [25,26] as supporting traditional 802.11 b/g/n. In terms of low power consumption, Bluetooth 4.0 known as Bluetooth Low Energy (BLE) is superior to traditional Bluetooth when exchanging small amount of data at high rates.

The IoT Smart devices benefit from a wired or wireless connection. According to S. Al-Sarawi et al. [27] in order to connect smart devices, there are different wireless communication technologies and short-range standard network protocols such as Internet Protocol Version 6 (IPv6), over Low power Wireless Personal Area Networks (6LoWPAN), ZigBee, Bluetooth Low Energy (BLE), Z-Wave, and Near Field Communication (NFC).

In order to connect the IoT device as local networks or individual IoT devices, different wireless communication technologies can be used, like Bluetooth and Wi-Fi variants:

Bluetooth is a protocol widely used for IoT application. According to Samie F. et al. [28], Bluetooth can be divided into the following categories:

- Classic Bluetooth suitable for data stream applications, which offers a high throughput and bandwidth, but with limited number of nodes in the network;
- Bluetooth Low Energy (BLE), known as Bluetooth smart has the advantage of a lower power consumption, lower setup time, and unlimited number of nodes and it is designed for short-rage, low bandwidth, and low latency IoT applications;
- Bluetooth 5.0 (BT v5) doubles the speed of low energy connections.

Wi-Fi is also another protocol used for IoT application. The Wi-Fi communication technology can be classified as such [28]:

- Conventional Wi-Fi (IEEE 802.11 b/g/n): A high energy consumption makes it unsuitable for ultra-low-power IoT devices, but with the advantages of a high bandwidth;
- Low-power Wi-Fi (802.11 ah) or HaLow: Suffers less interference, is able to reduce the energy consumption and to extend the range of transmission with less data rate for IoT applications.

Due to the fact that in the IoT field the wireless and battery powered sensors must be connected to the cloud, the need for long-range and low-power communications with Low-Power Wide Area Networks (LPWANs) protocols and technologies arose [29]. 

Although eHealth systems including wearable and ambient sensors may successfully contribute to elders’ quality of life especially in the context of NCDs [30], the power dissipation and cost of the IoT sensor nodes are not always taken into consideration in the specific IOT eHealth market even though these aspects may represent essential advantages for users in terms of handling operations, efficiency, and taking off [31].

The low-cost eHealth sensors require post-processing of data using filtering algorithms that eliminate acquisition errors without incurring long latency. Several filtering methods are used in the literature including Mean Average (MAW), Median Filter (MF), Kalman Filter (KF), Principal Component Analysis (PCA), etc. The filter parameters must be adapted according to signal acquisition and the specific measurement characteristics of biometric signals to be extracted.

As eHealth solutions represent a good candidate for monitoring patients, especially elders from the point of view of biometric data related to NCDs and/or MCIs, the current paper is targeting the architecture design, system implementation, and integration at prototype level based on COTS platforms along with optimizations for reaching a low-cost, low-energy, communication-efficient eHealth system. The main envisaged targets of the current work are the following:

Target 1. To define, implement, and integrate an eHealth system architecture and System-of-Interest (SoI) for monitoring biometric information of elders with NCDs and/or MCIs in independent living via Systems Engineering Methodology, based on commercial platforms (MySignals, Arduino).

Target 2. To define and design the mechanical case prototype of the eHealth SoI fitting the elders needs in independent living.

Target 3. To define and implement from scratch a cognitive assessment sensor in terms of choice reaction time as cognitive assessment tool and a corresponding measurement methodology definition.

Target 4. To optimize relevant COTS biometric sensor data (just the relevant sensors given the literature statement of cognitive decline and NCDs potential connection as referred in Section 1) by the usage of filtering algorithms.

Target 5. To analyze and optimize the power consumption of SoI.

Target 6. To optimize the short- and long-range communication latency for biometric data transmission locally and remotely to caregivers’ support.

The eHealth system presented in the herein paper is based on the low-cost eHealth Platform from Libelium, namely MySignals Hardware (HW) Development Platform—eHealth and Medical IoT Development Platform for Arduino [32], as opposite to the more expensive The MySignals Software (SW) Development Platform [33], based on an eHealth Sensor shield compatible with Arduino and several wired/BLE biometric sensors. 

The proposed SoI brings about a novel scientific concept and the integration of a low-cost COTS eHealth system with software improvement, electronic adaptations, and mechanical case prototype, centralizing specific NCD related biometric sensors together with an inhouse cognitive assessment tool for exploiting the NCD and MCI relation among elders. Thus, the present paper is achieving the targets already stated by approaching an exhaustive description of the system architecture and prototype implementations.

Besides the benefit of being used by elders, supervised by their caregivers, the proposed eHealth system is highlighting the avenue of deployment within three pilots in Romania, Italy, and Hungary, where NCD and MCI related data will be stored and classified in the project cloud to explore correlations and predictive analysis on the structured and unstructured data, with potential in elaborating MCI countermeasures for elders in terms of wellbeing recommendations. The pilots, implicitly the system demonstration together with end-users, are for the time being delayed given the COVID-19 pandemic, but the authors are trying to overcome the lack of access to primary end users (i.e., elders) via co-creation online meetings. Given the uncertainties provided by the pandemics and delaying the physical meetings and pilots, the authors succeeded to provide specificity on the eHealth concept, as accepted virtually by the end-users, in terms of: Firstly, taking into consideration the wider needs of elders in terms of NCDs for the reason they cannot physically interact with their corresponding General Practitioners; and secondly, providing them with support in terms of monitoring health in the high spread of SARS-CoV-2 (e.g., temperature, pulse oximetry, etc.). Hence, in Section 3, the concept and the eHealth system architecture, software implementations, and Hardware adaptations on COTS Platforms are presented, as well as the mechanical prototype CAD (Computer-Aided Design) concept for housing the system. According to Systems Engineering (SE) Methodology, the materials and methods are described for investigations into several perspectives, such as short-/long-range communication, data sensors accuracy based on filtering algorithms, and energy consumption measurements, whereas in Section 4, the results on the perspectives are presented. Section 5 draws up the main conclusions and perspectives in the realm of eldercare to elders with NCDs and/or MCIs.

## 3. Materials and Methods

The framework is pursued according to Systems Engineering Methodology (in compliance with International Council on Systems Engineering-INCOSE; European Cooperation for Space Standardization-ECSS), namely beginning with the end-user needs and the solution neutral function of the foreseen eHealth System-of-Interest (SoI) and continuing with the concept, architecture, proof-of-concept, and prototype along with verifications and validations [21,22].

### 3.1. Proposed eHealth System Concept and Architecture Definition

**Figure 1:**
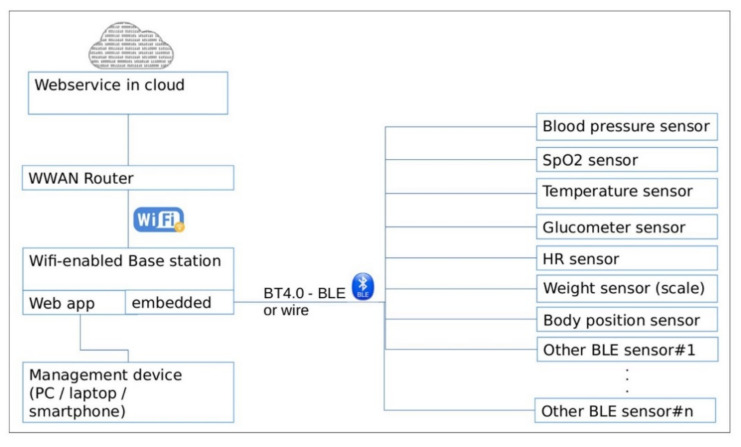
eHealth System-of-Interest (SoI) conceptual perspective.

In this context, taking into consideration the fact that the achievement presented herein stems from a Research and Development Project [34,35] within The Active and Assisted Living (AAL) European Programme, the proposed eHealth SoI is oriented towards the needs of elders suffering/being at risk of age-related NCDs and/or MCIs, in support to independent living in strong communication with a wide segment of stakeholders from volunteers and family members to professional caregivers and more. Under the “smart solutions for ageing well” vision fostered by the AAL Programme, the elicited solution neutral function of the eHealth SoI is shaped by acquiring biometric information from elders and transmitting it to the main stakeholder group mentioned previously, the latter with capability in handling and acting accordingly on the received information. The proposed SoI concept, as seen in Figure 1, comprises short- and long-range communication protocols as a Wi-Fi and Bluetooth4-enabled therefore scalable base station that offers two services:

- Embedded C++ application: Offers connection and readout of biometric sensors (e.g., the blood sugar, blood oxygen level, blood pressure, heartbeat rate, temperature, CRT sensor, etc.) [36]. It shares data with the cloud via a webservice.
- Web application [37]: Allows management and configuration for the base station. This option was considered better than OS-dependent smartphone app (iOS/Android). It will enable configuring the specific sensor, triggering of a certain measurement, displaying the result of the measurement, and setting the connection parameters used for cloud interaction, from any device connected to the same network as the base station (PC/laptop/smartphone).

As settled at the level of technology, for complying with one of the main requirements of the European AAL perspectives in terms of interoperability and open interfaces for achieving a European market, the SoI concept is oriented towards Open-Source Hardware (OSHW) and COTS eHealth Platforms including COTS biosensors. The decision was also made considering other criteria, such as lowering costs and increasing scalability and interoperability.

**Figure 2:**
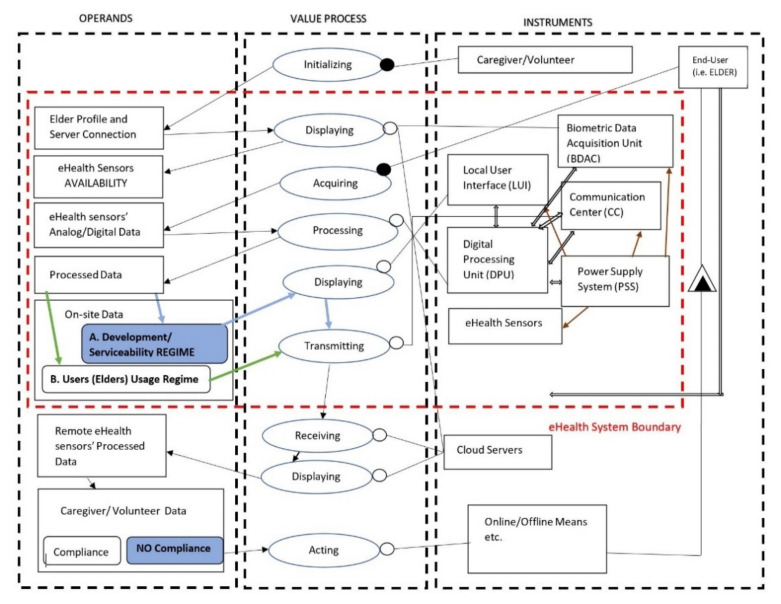
Object-Process Diagram (OPD) for the eHealth system architecture.

By identifying and decomposing primary and secondary value functions of the eHealth SoI together with the elements of form that sustain them, the system architecture is emerging via Object-Process Methodology (OPM), where the structural, functional, and behavioral views in a single coherent architecture are modelled [38]. Hence, in Figure 2, an Object-Process Diagram (OPD) for the eHealth SoI is shown where the specific relation of elements of form in terms of building blocks are linked to the processes and operands for achieving the intended system emergence and functions, as well notifying the system boundary, accompanying systems, and interfaces.

Based on the eHealth system architecture OPD view, the emergence, as the desired goal, suggests the primary value functions as automatically collecting biometric data in a non-obtrusive approach (e.g., unique physiological, physical, behavioral data) from elders at their dwelling place and further transmitting the data to specific caregivers/volunteers according to communication protocols established for the proper decisions of elders.

Having an in-depth analysis on system architecture OPD, the value pathway starts when the system is initialized by the second target user (volunteer/caregiver) with the elder’s profile and server network connections of the elder’s dwelling-place. Further, when the elder is properly using the system, there is the display function for elders exclusively in terms of eHealth sensors availability, the sensors being grouped in a list on the screen. The elder is able to acquire biometric data, the data are processed by the system, and two additional scenarios can occur in parallel:

- In the first regime of system development and serviceability, the biometric data are displayed and further transmitted via the internet exclusively to Information and Communications Technology (ICT) development teams. In the second regime of daily usage by the elders, the biometric sensor data are not displayed on the unit screen, just transmitted to the internet to caregivers/volunteers outside the eHealth system boundary for specific decisions to be made. The second regime stems from co-design performed sessions, as required by stakeholders. Thus, the system may accommodate changing needs, fitting the scenario of internet absence and communication failure for elders if the two regimes will be combined.

Considering the form aspects, namely instruments along with structural relationships among them, as illustrated in OPD system architecture, the functional blocks identified and defined by specific system requirements are the following:

- Biometric Data Acquisition Unit, which must integrate COTS OSHW components for data acquisition in digital and/or analog format, data processing, and transmission using analog-to-digital converters, multiplexers, standard communication protocols, etc.
- eHealth Sensors that could be presented as a customizable biometric sensors package for the primary target group (i.e., elders). According to the system requirements, the considered biometric sensors are: Blood pressure sensor, pulse oximetry sensor, body position sensor, temperature sensor, glucometer, spirometer, body weight scale, and CRT sensor for cognitive assessment. The acquired data by the e-Health central unit from the sensors (intended to be used individually) are collected, processed, and automatically sent to the Cloud.
- Power Supply System (PSS), which must provide with power autonomy the eHealth system with the screen ON and all sensors in operating condition. The user must be able to charge the system while it is in use; the system must function in “always-on” mode and not enter in standby mode; the e-Health system is turned OFF using an ON/OFF button.
- Digital Processing Unit (DPU), which must communicate to transmit information between the Communication Center, Local User Interface, Biometric Data Acquisition System, and Power Supply blocks.
- Client–Server Communication Unit uses the network card Wi-Fi System-on-a-Chip (SoC) in order to send information to the Cloud server, and the system will communicate via Wi-Fi with the user’s home wireless router. The system is intended to communicate with the server using an encrypted channel with HTTP protocol and secured with SSL/TLS certificates, to send data only when it has real values recorded from the sensors and at a configurable period of about 10 s.
- Local User Interface (LUI) must display locally the biometric sensors availability, if it has found at least one Bluetooth-enabled sensor and the sensor pairing. The system, according to the system architecture, will pursue the parallel functional regimes described above.

Further on, as it is presented in the following sections, the prototype implementation and integration were carried out starting from MySignals Hardware (HW) Development Platform—eHealth and Medical IoT Development Platform for Arduino [32], which is an eHealth Sensor shield compatible with Arduino and several wired/BLE biometric sensors. Based on [32], different technical trade-offs were done in order to accommodate a proper board and microcontroller, a mechanical prototype case targeting end-users’ operations in their dwelling places was elaborated, such as improved short (i.e., new BLE manager development) and long (improved Wi-Fi driver with decreased Wi-Fi data latency) range communication protocols, increased biometric sensors accuracy by applying different filtering algorithms, and power consumption investigations in different operational scenarios.

Even if IoT wearable sensors and devices are abundantly emerging on the market, elders’ reluctance to technology together with their acceptance for this type of technology rise significant challenges for developers. During the co-design sessions, authors identified these facts, therefore the choice of IoT sensors and devices used in the proposed eHealth system was not trivial.

### 3.2. Electronic Architectures and Trade-Offs

The current subsection presents a hardware and software perspective on integration and implementation of the eHealth SoI, especially considering the functional blocks defined in the previous system architecture section. The current section presents the hardware adaptation considering the Arduino OSHWs trade-offs (resulted in the configuration composed by Arduino Mega2560 board, Libelium HW shield and ESP8266 wireless SoC) and software implementations. 

**Figure 3:**
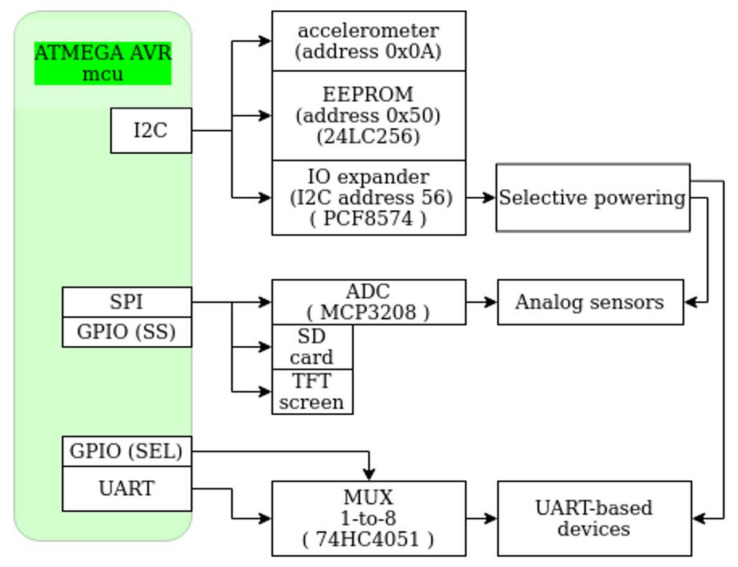
MySignals Hardware (HW) block schematic [EHB2020]. The ESP8266 Wi-Fi card is an Universal Asynchronous Receiver-Transmitter (UART)-based device.

Biometric Data Acquisition System was developed implementing the Libelium eHealth COTS platform (Figure 3), a development board that allows connecting and processing data collected from biometric sensors. More precisely, the platform consists exclusively of a shield with inputs/outputs for Libelium-branded sensors. Based on this platform, a whole integration process of hardware and software implementation was implemented coherently, as it can be analyzed in the following sections.

The platform can integrate biometric sensors (pulse oximeter, body thermometer, body position, blood pressure, spirometer, glucometer, body scale) connected wirelessly via Bluetooth—Bluetooth Low Energy (BLE), or Universal Asynchronous Receiver-Transmitter (UART); some analog sensors may be additionally connected via the existing eight channels provided by the on-board Analog-to-Digital Converter (ADC) (depending on required signal conditioning/gain). It should be mentioned that the development board supports analog sensors with a maximum of three channels (i.e., digital body position detection sensor that acquires information through four wires: Three axes of the accelerometer component and a Ground reference connection).

The Power Supply System (PSS) was implemented as an internal Li-Po rechargeable battery—Power Bank Voltaic V50 (capacity of 47 Wh) with an autonomy of about 47 h of online system usage (maximum time usage: 77 days when eHealth system is off, but having Voltaic’s always-on mode activated). The battery may be recharged from a 230VAC to 5VDC at minimum 2A PSU in around 5 h; the eHealth system may be used normally while charging. The entire eHealth system has an average power consumption of 120 mA—with the screen’s backlight off and 200 mA—with the screen’s backlight on (the backlight can be turned on or off programmatically) in default use-case. 

Digital Processing Unit (DPU) was designed as a logical system that is based on the processing capacity of the Arduino development board used in the eHealth Unit configuration. Data collected from analog sensors and/or digital devices is usually in a raw (unprocessed) format, therefore requiring digital filtering;

The Wi-Fi connection is provided by the ESP8266 SoC, using UART interface configured at 115,200 bps. The default AT-command stack was found to be very inefficient (at least eight commands needed to be used for successful data transmission) and it was replaced by a custom-designed firmware [39] (modified code is hosted on [39]) to allow hotspot-SSID (Service Set Identification) configuration and single-command JSON-format HTTP-PUT (Hypertext Transfer Protocol-PUT) requests. The server receives the data, processes them, stores them, and displays them in an Admin Center application.

Three boards are stacked: The base board Arduino (connected via USB for power and debug), the MySignals HW PCB and the ILI9341-powered display PCB.

A Proof-of-Concept (PoC) system [35] was developed using the open-source hardware development board Arduino UNO R3 (ATmega328P) with the Libelium MySignals shield as shown in Figure 3. The PoC system was initially implemented using two sensors (an analog one and a BT-based one, to ensure a certain confidence in estimating the required program memory size). The program memory was found to be too small (totaling 32 kB, of the currently needed 31 kB), so we ported the main app’s code to a 48 kB-long program memory included on-Arduino UNO Wi-Fi Rev2. Unfortunately, this platform was abandoned afterwards when full documentation for BT sensors was not provided by the manufacturer. As a final solution in the eHealth system prototype development, we switched to the Arduino Mega (containing ATMEGA2560 with 256 kB of program memory).

The utilized program memory for the ATMEGA2560 MCU of ArduinoMega board, was around 66 kBytes (including the blue-white background bitmap) for all the desired sensors, including software code for BLE communication, display, data storage, network configuration, and Wi-Fi connectivity to the Internet using custom AT command towards the Wi-Fi card.

In Table 1, one can see the number of Bytes, each software module takes inside the program memory of the ATMEGA2560 MCU.

The acquisition data rate of the ADC was set to 15 kSa/s and its resolution to 10-bit for any channel (channels are not sampled in parallel: There is only one ADC peripheral in ATMEGA2560, and the analog inputs are multiplexed). The generated data size is 15 k of 10-bit per second. These data are postprocessed (in embedded), and a representative value is sent into the cloud, reducing the required bandwidth for the network card.

Important code sections have been redesigned and rewritten, reorganized modularly, with the effect of considerably increasing the operating efficiency of the eHealth system. The code used for the eHealth Unit and peripherals (biometric sensors, biometric devices, display, etc.) has been improved and extended (for example CRT sensor) so that the entire embedded system exceeds the initial performance previously described by MySignals in terms of significantly lower program memory (see Table 1) and operation of the acquisition system at a higher speed. 

**Figure 4:**
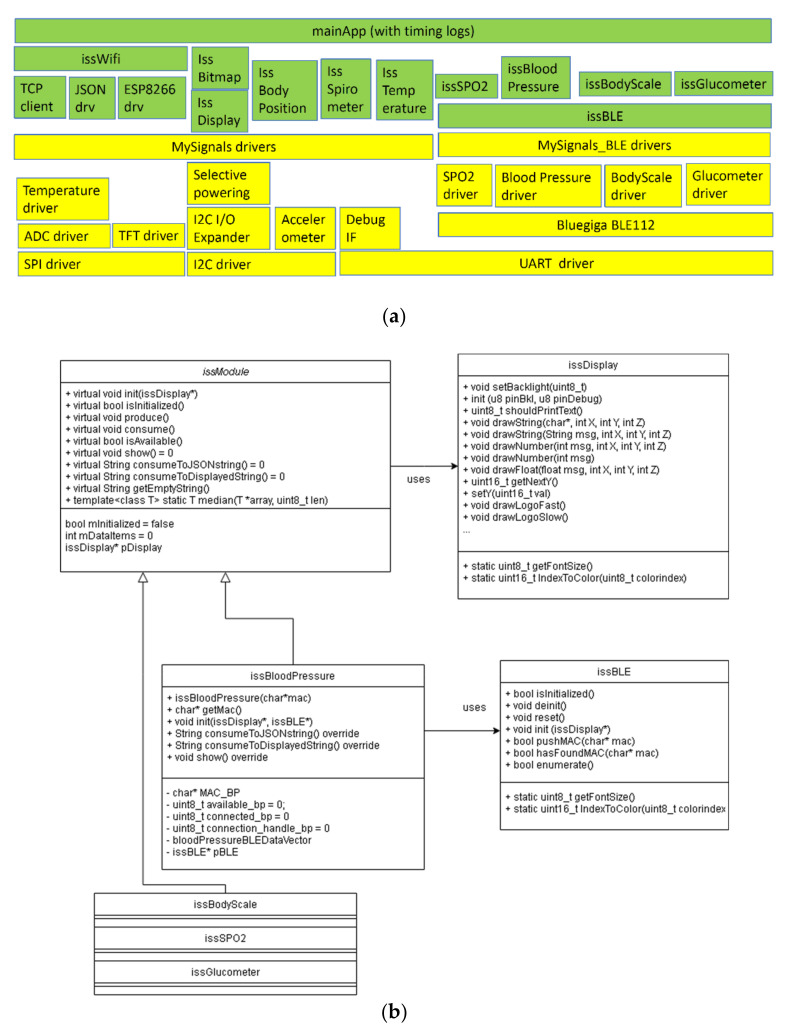
(**a**) The software stack block diagram. With yellow: Drivers already available as separate applications, in MySignals examples. With green color: High-level drivers developed by authors. (**b**) The software’s (partial) class diagram highlighting public (+) or private (-) members or methods.

Software architecture was developed to mirror the hardware components (one low-level driver, one high-level driver, and the app on top of it all). Figure 3 shows a block schematic of the hardware used in the eHealth platform, and Figure 4a shows the corresponding software modules developed for this system.

In Figure 4b, the partial class diagram of the software is drawn. All sensors’ drivers are inherited from issModule and use issDisplay. Additionally, all BLE-enabled sensors use the issBLE class.

In terms of Software implementation, it is schematically split in two parts as follows.

Basic Embedded Software. At the present moment, the eHealth system is using MySignals configuration, Arduino Mega, and an ESP8266 network card for reasons related to high program memory consumption (64+ kB); this is around 25% of maximum capacity and will facilitate software and hardware development perspectives.

**Figure 5:**
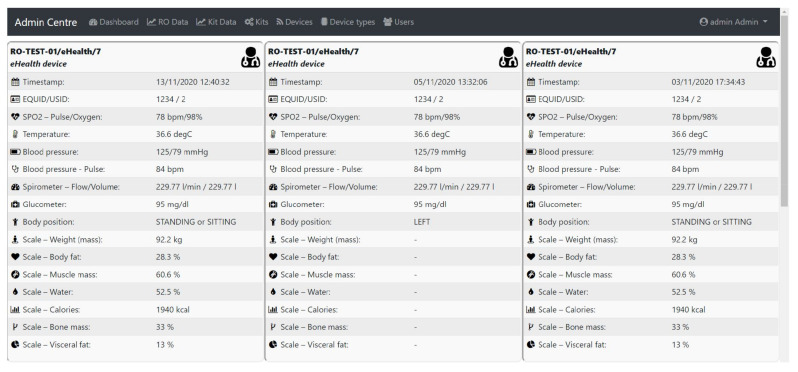
SAVE Cloud interface for Admin Centre showing last 3 measurements (in the middle one the scale was not used).

Additional Embedded Software Modules. eHealth Unit system benefits from a series of global software improvements that can be presented modularly in terms of results:

- Better Bluetooth manager (by registering the MAC addresses at boot time and specific search of the devices by the MAC address later on).
- Better Wi-Fi driver (data communication between eHealth Unit and SAVE Cloud regulated to communicate minimum required data: One command containing server’s IP, port, endpoint, and payload).
- Added brightness sensor (in order to control the brightness of the screen).
- Digital signal filtering: Some sensors require data filtering in order to eliminate certain specific artifacts of the acquired signal, e.g., temperature sensor induces a noisy signal—the solution found was to apply a median filter.
- Software Relationship: eHealth System to Cloud. Currently, the data transmission to the SAVE Cloud, which ensures synchronous viewing of collected data (see Figure 5), has been successfully completed and the data transmission format has been established as a JSON template based on JavaScript principles. Prior to this step, the data transmission between PC and server was implemented via same HTTP POST requests on a locally node.js server-side JavaScript application. Finally, tests and demonstrations were performed to observe the operation of the system in real cases.

For reasons of available program memory, ATMEGA2560 was chosen as a main MCU. The prototype therefore uses the configuration composed by Arduino Mega2560 board, Libelium HW shield, and ESP8266 wireless SoC as network chip (on a ESP-01 PCB) connected via UART interface.

### 3.3. Mechanical Prototype

The case concept rationale is given by the following main features:

- High-touch case concept, referring to the involvement of elder’s attention and trust in the system; the concept tries to build a connection between the elder and the technology in terms of handling operations.
- Easy access to biosensors, referring to the natural movement for the elders, namely pulling and pushing the drawers for accessing the biosensors;
- Simple sensors repository and natural approach of handling, given the fact that the repository looks like drawers, where sensors are just introduced in compartments without complicated grips or interfaces.
- Robust design, where the design is related to a traditional chest-of-drawers, just at a smaller scale.
- Ergonomic control panel for elders, given the existence of an on/off button on the top near a Thin Film Transistor (TFT) screen listing the biometric sensors’ availability.

**Figure 6:**
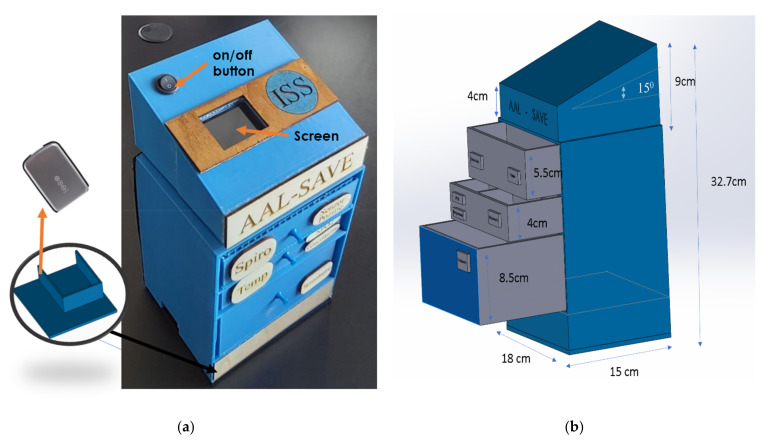
Device assembly incorporation: (**a**) Physical prototype and (**b**) 3D model design.

The mechanical design of the assembly was developed using SolidWorks CAD software (Dassault Systèmes, Vélizy-Villacoublay, France) following several meetings that took place in order to establish the concept. Thus, it is structured in the form of a tower with drawers and the component devices incorporated, as seen in Figure 6.

**Figure 7:**
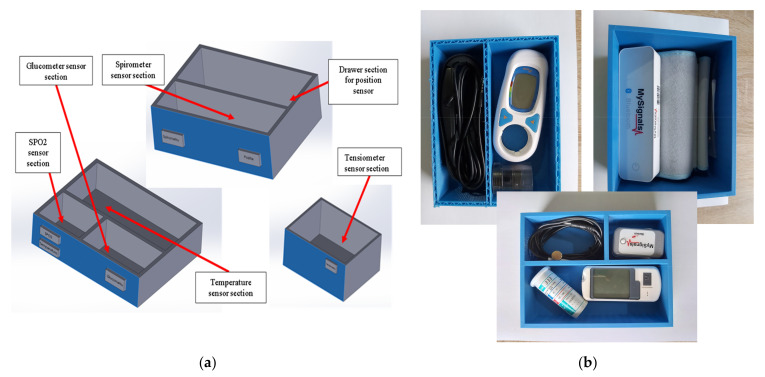
Drawer sections for device storage (**a**). Libelium biometric sensors in photos (**b**).

Each system component has its own place, especially designed and marked in order to facilitate the use and storage of the devices (as shown in Figure 7). The “chest-of-drawers” concept with labels on the drawers indicating the sensor location stem from co-design physical interactions with the end users (especially elders), therefore the eHealth mechanical concept is as required on behalf of elders, whereas technology acceptance is positive. The component situated on the top, which incorporates the screen, was designed at a 14-degree angle so the user can easily read the data shown on the screen and the battery is placed in the lower part in order to allow easy access to charging ports.

### 3.4. Short and Long Range Communication Latency

The ESP8266 network card was used for Internet connectivity. It boots initially in AP-mode unless it can connect to a Wi-Fi network previously used. It then enters normal operation as a network component that provides wireless data service. Regarding range of communication and consumed power, the following report (Table 2) resides in a University internal study, unpublished yet. 

### 3.5. Analog Sensor’s Processing and Accuracy

Literature overview on biometric signal filtering methods reveals a study [40], similar to the approach herein using (the default) Arduino UNO board and MySignals HW—eHealth and Medical IoT Development Platform for evaluating the main physiological parameters (i.e., body temperature, EMG, BP, SPO2, blood glucose, EKG) of the human body. The authors use a routine of amplifying, measuring, and filtering the data collected from the sensors using a python software application.

Another comprehensive work [41] considers enhancing the accuracy of their low-cost body sensors through the usage of signal filtering algorithms. The study addresses an extensive approach in evaluating certain filtering methods as Moving Average Window (MAW), Principal Component Analysis (PCA), and Kalman Filters (KF). The authors evaluated the filters performance having as reference the signals provided by certain certified sensors (i.e., body thermometer and GSR sensor) and concluded, by comparing the Mean Square Error (MSE), the Normalized MSE (NMSE), and the Signal-to-Noise Ratio (SNR), that the MAW filter applied on five samples provides the most significant result followed closely by PCA used rarely in the literature for noise reduction of the biometric signals. On the other hand, the work revealed that KF is not appropriate for filtering body thermometer and GSR sensor data as it returned higher errors.

Also, the smoothing EWMA (Exponential Weighted Moving Average) filter [42] is used in another study for certain e-Health sensors. Band pass filters (BPF) have been used for filtering EEG signal [43], KF for Physical activity tracking, and Hidden Markov Models (HMMs) for periodical movements [44].

The targeted eHealth sensors are the pulse–oximeter and the body thermometer.

Given the paper perspective fostering the potential exploitation of centralizing noncommunicable disease (NCDs) biosensors and cognitive decline CRT [6,7,8,9,10], the targeted sensors mentioned above were effectively measured and processed given the following: These corresponding physiological parameters (such as pulse rate, oxygen saturation, temperature) are related to cardiovascular diseases and pulmonary diseases, and also further sought as support in monitoring health in the high spread of SARS-CoV-2 virus, as stated and required by the SAVE project stakeholders; the rest of the sensors, even though they are important according to stakeholder requirements, provide either filtered processed signals (blood pressure sensor, glucometer) or maximum value (spirometer, weight) once per measurement cycle.

In Section 4, a qualitative evaluation of the biometric data filtering methods will be presented in accordance with the methods investigated in the literature and applied to the biometric signals collected by the eHealth prototype.

Physiological data used in this study are real, collected from a male human subject, aged 35–40 years, without any known medical problems. The study was deployed in laboratory conditions based on experimental methodologies with respect to biometric devices utilization (i.e., body thermometer, pulse oximeter).

Regarding the experimental methodology, there were two scenarios defined according to temperature and pulse oximetry measurements. In the former scenario, the subject holds the temperature sensor tight inside the left-hand fist for a period of 50 s after which he releases it; in the latter scenario, the pulse oximeter is worn during a session that includes three rounds of moderate effort and three rounds of rest pursuing fitness activities and break in standing conditions.

The fingertip pulse-oximeter instantly communicates the data acquired (i.e., pulse rate and blood oxygen saturation) over a BLE wireless connection. The body thermometer measures the skin temperature and communicates with the eHealth Unit via wire transmitting an analogue signal. Three eHealth signals will be considered consequently further on: One Temperature log that lasts 120 s with a sampling rate of 65 acquisitions per second and two logs that lasts 275 s, collected with a sampling rate of 8 acquisitions per second. In Section 4.2., the data acquired from two of the eHealth sensors will be processed in order to enhance the signal properties using several filters approached in the literature that are described theoretically further on. 

The Moving Average Window (MAW) Filter is often used to smooth the signal by averaging a predefined set of input samples of the data array. The MAW Filter introduces a delay due to the computational effort that is proportional to window length and can be removed. The MAW filters a sequence of n elements: $x_{1}$, $x_{2}$..., $x_{n}$ using a window characterized by size parameter *k* > 0, where $k^{th}$ moving average of the given sequence is defined in (1–3) as follows:

(1) $$y_{1}=\;\frac{1}{k}\;\left(x_{1}+\;x_{2}+\ldots +x_{k}\right)$$

(2) $$y_{1}=\;\frac{1}{k}\;\left(x_{2}+\;x_{3}+\ldots +x_{k+1}\right)$$

(3) $$y_{n-k+1}=\;\frac{1}{k}\;\left(x_{n-k+1}+\;x_{n-k+2}+\ldots +x_{n}\right)$$

EWMA (Exponential Weighted Moving Average) is a variation of moving average filter characterized by weighting factors that decrease exponentially with respect to the definition (4):

(4) $$y\left[n\right]=\alpha \sum_{k=0}^{n}{\left(1-\alpha \right)}^{k}\;x\left[n-k\right]$$

where *y*[*n*] is the current value, *y*[*n* − 1] the previous value, and *α* ∈ (0,1). 

Kalman Filter (KF) requires low computational power and provides estimates of variables taking into account the measurements observed over time. KF provides an estimate of position and velocity taking into account the previous estimate for each sample of signal x with respect to the Equation (5):

(5) $${\hat{X}}_{k}=K_{k}\cdot Z_{k}+\left(1-K_{k}\right)\cdot {\hat{X}}_{k-1}$$

where $Z_{k}$ is the measured data, ${\hat{X}}_{k-1}$ is the previous estimate, and $K_{k}$ is Kalman gain. Hence, KF uses for each state the best average factor and takes into account estimates of the past signal.

The Median Filter (MF) is used in order to remove noise by sliding a predefined window over the data array. The MF is preferred for removing noise in digital signals with good results in terms of smoothing the regions of the signal but affecting edges. In addition, the MF is used for digital processing where speckle noise and salt-and-pepper noise are common. The 1D median filter replaces the central value of the window by the median value in (6):

(6) $$y\left[1,m\right]=median\left\{x\left[i\right],\;i\in w\right\}$$

where w represents a neighborhood $\left[1,m\right]$ of the vector.

### 3.6. Control Circuitry and Power-Consumption Investigations

**Figure 8:**
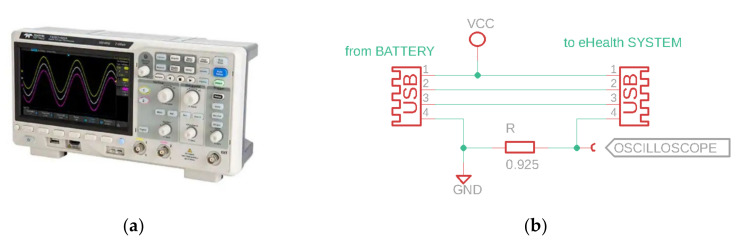
The T3DSO1302A oscilloscope used in current measurement (**a**); the current-measurement rig (**b**).

The current measurements were performed using the T3DSO1302A digital sampling oscilloscope from Teledyne/LeCroy, by measuring the voltage drop on a low-side shunt as seen in Figure 8. Two channels were used with the stock T3PP350 passive probes: CH1 for triggering on UART3-TX and CH2 for actual current measurement (voltage drop on 0.925 Ω resistor). Logfiles were saved as 3-columnMatlab (Mathworks Inc., Natick, MA, USA) friendly file format (timestamp, channel1_amplitude, channel2_amplitude). Sample rate was set to maximum 1 GSa/s for all measurements smaller than the available 1.4 Mpoints oscilloscope memory (the only one longer than that, was the display initialization measurement, where we had to drop the sample rate to 50 MSa/s). The 1 GSa/s sample rate was used to catch high-speed variations in current consumption of the Wi-Fi SoC (ESP8266EX) running up to 160 MHz.

### 3.7. Choice Reaction Time (CRT) Measurement Methodology and Sensor Implementation

Starting from the premise that reaction time to visual stimuli increases with age [45], especially in terms of chromatic light flashes discrimination [11,46], the Choice Reaction Time (CRT) methodology in this initiative is based on a visual CRT in a specific paradigm based on several visual stimuli and two response buttons. 

The methodological principle in this study resembles with the one in the [10], where it was designed a target letter to be responded by pressing one button, whereas distractor letters to be responded by pressing the second button.

Thus, the present methodology imposes a trichromatic RGB (red, green, and blue) light-emitting diode (LED) stimulus with the following procedures: The blue LED color represents the target, and when the blue stimulus lights up, the elder has to discriminate, select, and execute the right button (R); the red or green LED colors represent distractors and when the red or green stimulus lights up, the elder has to discriminate, select, and execute the left button (L). Physiological data used in this study were real, collected from a male human subject, aged 35–40 years, without any known medical problems. The study was deployed in laboratory conditions based on experimental methodologies with respect to CRT utilization.

**Figure 9:**
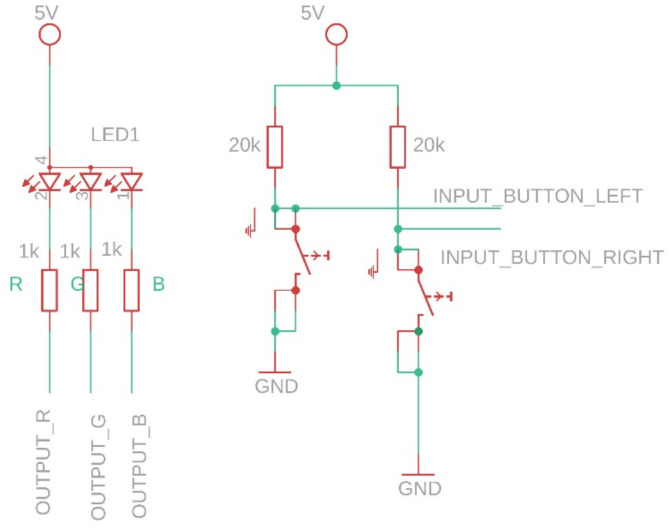
CRT sensor equivalent electrical schematic.

Regarding the CRT setup, the subject sits on the chair and manipulates the CRT device with both hands, supporting the device with the index fingers and pressing the two buttons with the thumbs. As in [10], stimulus duration and stimulus onset asynchronies (SOA) are set at 200 ms and 2500 ms, respectively. If the elder presses the correct button for the respective led (Right for Blue, and Left for Red and Green) the timer stops and the stimulus-to-response time interval is logged; otherwise, the timer continues running until stop condition occurs (correct button is pressed or 2500 ms have elapsed since last stimulus occurrence). The whole experiment lasts 5 min. The measurements are performed as in-house testing for methodology and sensor verification and validation. The CRT sensor is comprised from two tactile switches and one 5 mm RGB-LED. The LED segments are CA (common anode) and the current through them is limited by the 1 kΩ resistors (Figure 9). The tactile switches put microcontroller’s inputs to ground whenever they are pressed. The microcontroller’s switch inputs have the weak pull-up enabled.

**Figure 10:**
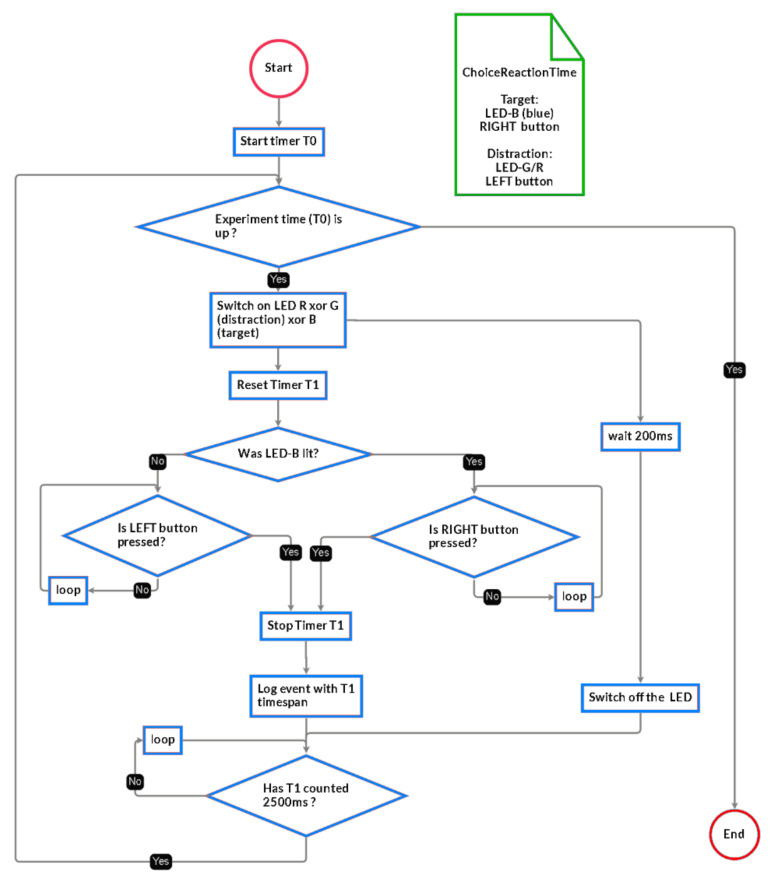
Methodology for the CRT measurements.

The state machine implementing the measurement is described below (Figure 10).

Authors performed a SRT test (1 stimuli, 1 button) and found out that running the test in embedded provided an average of 220 ms with a standard deviation of 70 ms, whereas the running of this test on the computer indicated 607 ms average and similar 70 ms standard deviation. The conclusion was that the extra time was wasted on the route from the mouse to the PC, through all the hardware and software stack, into the preemptive non-real-time OS, and finally into the high-level application.

## 4. Results

### 4.1. Control Circuitry and Power-Consumption Investigations

**Figure 11:**
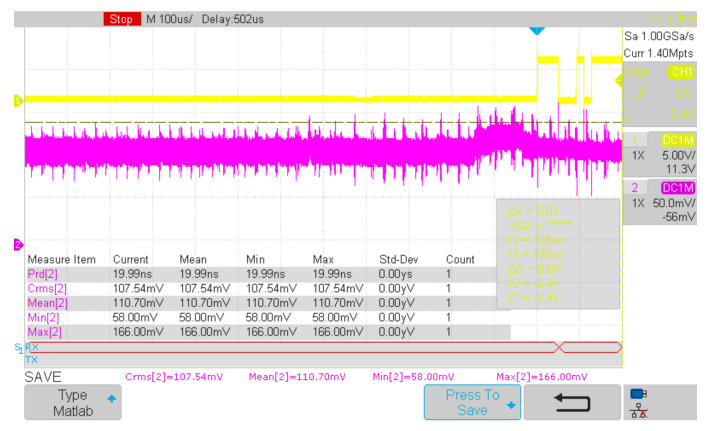
A sample snapshot from the oscilloscope, triggered on CH1 (CH2 shows the current, that is, the voltage drops across the R resistor in Figure 8b. The highest icc consumption in this picture can be computed as: 166 mV/0.925 Ω = 179.45 mA.

Power-consumption investigation (setup and procedure):

Required hardware: The Arduino MEGA2560 baseboard with a MySignals Hw shield and ESP8266-based network card, oscilloscope [Figure 11, PC Required software: Arduino IDE, the embedded application [36], which adds triggering and timing measurements was uploaded on the ATMEGA MCU. The PC application [47] used to process the acquired data.

Procedure: An oscilloscope was connected to the CH1 as trigger, tied to UART-TX4 line of the ATMEGA chip. CH2 monitors the voltage drop on shunt resistor (see Figure 8b). The oscilloscope is armed then the application [36] is uploaded into the MCU. The internal logfile with time measurements is printed in the UART console: Copy this content into a file1. Save the oscilloscope trace onto a USB stick as Matlab-compatible format file2. Run the PC application [47] on these files (file1 and file2) and get the stats output (Table 3).

In the figure above (Figure 11), triggering is made on CH1, when a byte is sent via UART3-TX. The current channel (CH2) measures the voltage drop on a low-side resistor of 0.925 Ω. Two logs are extracted and synchronized using a PC C++ application [47]: The one from the oscilloscope, and the time-log kept in the RAM memory then sent by the eHealth system via UART at the end of benchmarking, after oscilloscope triggering, just as below:

- 22:07:48.130 → Init: 0 us to 284 us -> 284 (1 samples);
- 22:07:48.130 → disp.init(): 284 us to 6,567,184 us -> 6,566,900 (1 samples);
- 22:07:48.130 → disp.setBacklight(HIGH): 6,567,184 us to 6,567,216 us -> 32 (1 samples);
- 22:07:48.130 → disp.drawString(ISS app started!): 6,567,216 us to 6,567,292 us -> 76 (1 samples);
- 22:07:48.130 → disp.setBacklight(LOW): 6,567,292 us to 6,567,328 us -> 36 (1 samples).

In Table 3, we have measured the graphic driver in various scenarios (initialization, switched-on backlight, switched-off backlight, and drawing a string on the display). 

Wireless card improvements determination (setup and procedure):

Required hardware: PC, ESP8266 board (e.g., ESP-01 [48]) on a USB-to-UART board
Required software: Arduino IDE, ESP8266 embedded test application [49], node.js PC web-application [50] (as local cloud), python PC test scripts.
Test procedure (for improved version): Connect the ESP8266 board via USB-to-UART board to the PC. Enter boot mode. Program the [48] application into the Wi-Fi SoC. Reset. On PC, start the node.js web-based application [50], this will enable a listening TCP server, which will log the incoming data packets. Start the test by running the txFastTest.py script; it will output files similar to txFastResult.txt from [49].
Test procedure (for initial version): Similar to above, but change the Wi-Fi SoC application to default AT stack, and change the python script to run to txSlowTest.py
Extracted data returned by python scripts will present data similar to Table 4 and Table 5.

We compare below (Table 4) the improvements made in changing the AT-command stack of the ESP8266 802.11 chipset. A speed comparison is made by sending the same 189-bytes data packet to a locally implemented cloud. Statistics were extracted from 3600 packets.

In Table 5, one can see the old stack time-spending; most of the time is wasted negotiating the data in the HTTP-PUT packets, between the host (AVR) and the wireless card. Statistics were extracted from 3600 packets.

### 4.2. Analog Sensor’s Processing and Accuracy

Filtering performance setup and test procedure
Required hardware: PC, Arduino MEGA2560 board
Required software: Arduino IDE, embedded application
Test procedure: Program the ATMEGA2560 microcontroller with the embedded application [36] (and enable the filtering tests—see main file). Read log printed in UART console on the PC.

**Figure 12:**
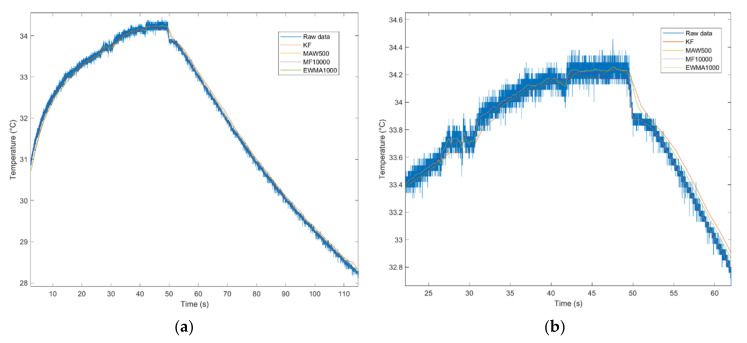
Temperature data filtered in original view (**a**) and detailed view (**b**).

**Figure 13:**
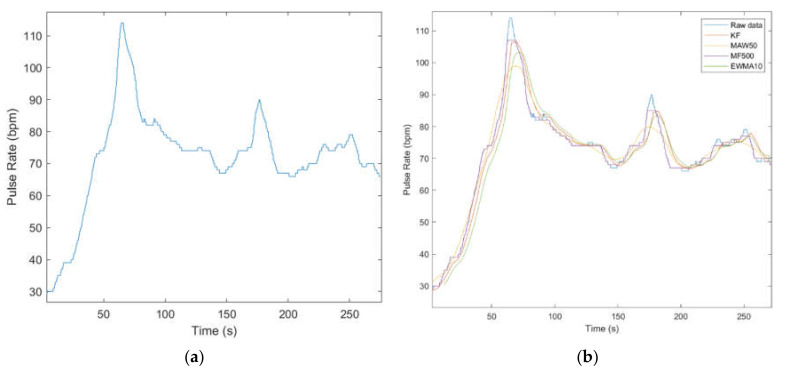
Pulse rate raw (**a**) and filtered data (**b**).

It is worth mentioning that Signal Filters may be used in different scenarios specific to each eHealth parameter to be measured taking into account the compromise between filters’ smoothing and/or signal details preservation features. For instance, in order to smooth noisy eHealth signals as body temperature or oxygen saturation (Figure 11), MAW and EWMA provide a good response. On the other hand, in order to filter eHealth signals where velocity and points of local minima and maxima are to be considered (i.e., temperature, or even betterpulse rate) as shown in Figure 12 and Figure 13 KF provides the expected response. 

According to the sampling rate of the signals, the following filters will be tuned with different parameters in order to achieve the expected filter response. In the following two images, KF is applied on 1000 frames decimated signal of temperature and 30 frames decimated signal of oxygen saturation and pulse rate.

Regarding the temperature signal evaluation scenario, the subject is asked to position the sensor in contact with his skin and after a period of time to remove it. The purpose of the measurement is to detect the global maximum of the signal. 

The authors considered applying KF on 1000 frames decimated signal, MAW on 50 samples window, MF on 500 samples window, and EWMA on 10 samples window. According to Figure 12, all filtering algorithms behave similarly, noting that KF manifests a delay in the first stage of the signal, which does not affect the temperature detection.

Regarding the pulse rate and oxygen saturation signal evaluation scenario, the subject is asked to place its finger between the two plates of the pulse oximeter. The device provides instant results already processed. In this paradigm, it is necessary for the data to be post-processed taking into account the acquisition errors caused by acquisition digital noise specific to this range of low-cost biometric devices. In order to gain its performance, it is desirable to filter and/or analyze regions of the signals acquired and eliminate non-compliant features (i.e., impulsive noise, salt and pepper noise, etc.). 

Given that the pulse oximeter device acquires 2 biometric signals (i.e., pulse rate and oxygen saturation), the authors considered the raw pulse rate data according to their acquisition requirements and that the data should not be enhanced by biometric signal filtering methods. On the other hand, the raw oxygen saturation data are noisy and biometric signal filtering methods must be applied.

As it can be observed in Figure 13, signal filtering can be bypassed in the case of Pulse rate filtering because it causes significant loss in signal amplitude despite the smoothing benefits.

**Figure 14:**
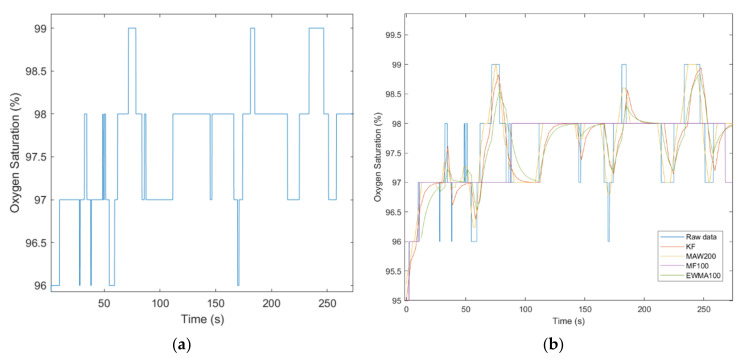
Oxygen Saturation raw (**a**) and filtered data (**b**).

Figure 14 shows off the low quality of the Oxygen Saturation sensing and the serious need of signal filtering. The authors applied KF on 30 samples decimated signal, MAW on 200 samples window, MF on 100 samples window, and EWMA on 100 samples window. The mean value of the Oxygen saturation vector is 97.6095%, and according to the authors, it is desirable to extract the long-term characteristic of the signal (i.e., MF100). Otherwise, in order to extract short-time features of the Oxygen Saturation, the authors may recommend KF, MAW200, and EWMA100. 

Further on, the authors implemented certain processing algorithms by an embedded controller unit and measured the processing time. Table 6 and Table 7 show time measurements for various window-size of moving average and median filters running on 16 MHz ATMEGA2560MCU. The devices (i.e., pulse oximeter is characterized by an acquisition rate of 0.1 s and a resolution of 1%, whereas the body thermometer may be sampled at up to 15 KSa/s). 

Column 4 shows the amount of time it takes the MCU to process an entire 1000 samples-buffer using windows of various sizes, whereas the fifth column shows the amount of time spent by the MCU to output one post-processed sample (equals to column 4 divided by number of windows).

Total samples considered (i.e., 1000 samples) are equivalent to about 120 s of acquired signal from pulse oximeter device (approximately eight samples per second) and 2 s of acquired signal from body thermometer sensor (approximately 1000 samples per second) requiring different signal processing approaches. The desired sample buffer for any sensor should cover up to 10 s (more than that would disturb the elder). The maximum amount of 8-bit samples the MCU can store in its 8 KB RAM memory is around 2000 (reserving some RAM space for the application static memory and stack). Given the maximum acquisition data rate for the SPO2, 80 samples are enough to cover 10 s, and for the temperature sensor—2000 samples will cover 2 s.

Hence, the body thermometer signal may be enhanced using large window-sized moving average (i.e., 1000 samples) considering the fact that moving average filtering is 10 times faster than median filtering and on the other hand, oxygen saturation signals may use median filtering as well as moving average filtering. However, the temperature sensor is recommended to be post-processed with 1000-sized window moving average filtering.

Taking into account the approached described above, the authors consider as minimum hardware requirements 8 kB SRAM (for the application + required memory for the filters) and a 10-bit A/D converter (for reading analog sensors, keeping only 8 bits for the 25 … 50 degrees Celsius range, with 0.1C resolution)

Filtering was implemented as much as possible in-place, to trade-off filtering speed over utilized RAM.

### 4.3. Choice Reaction Time (CRT) Measurements and Results

CRT measurement setup and procedure
Required hardware: ATMEGA board with CRT test fixture [Figure 12]
Required software: CRT measurement test application [51]
Procedure: program the embedded app (.ino file in [51]) in the ATMEGA microcontroller and open the UART console on the PC. Keep track on the R, G, or B led and press as fast as possible, either the left or right buttons (according to description of Section 3.7). A logfile is printed in the console, as a 4-column table: Timestamp, correct-pressed, color, reaction time. Process the data using the xlsx sheet in [51].

For the mouse reaction time, use a PC with mouse, and then run the Python mouse.py application in [51]

The CRT measurement was performed by generating pseudo-randomly stimuli events (swiching-on LED R xor G xor B for the RGB LED). The setup was described in Section 3.7.

**Figure 15:**
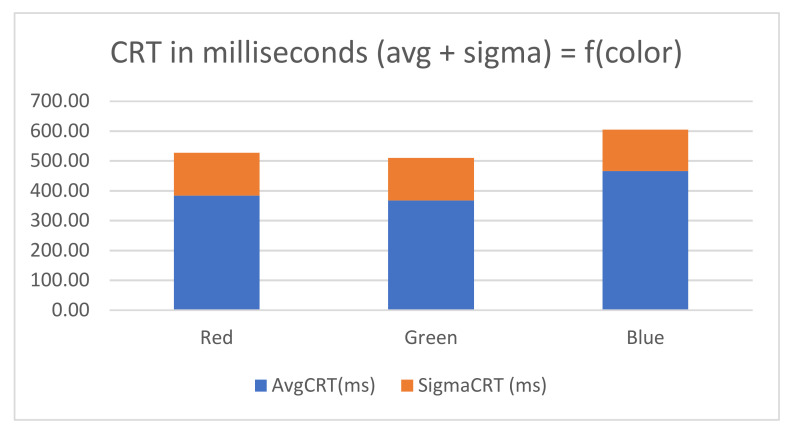
Average CRT and standard deviation (sigma) as function of color. Blue is target color, Red and Green are distractions.

The proportion of occurrence of these events were measured over two experiments of 5 min and are as shown below in Table 8:

The average CRT time for R and G colors is in range of 360–380 ms whereas the Blue is 466 (as seen in Figure 15). This might be due to the expectance that 66% of the time, the left button should be pressed, and only 33% of the time the right one. 

**Figure 16:**
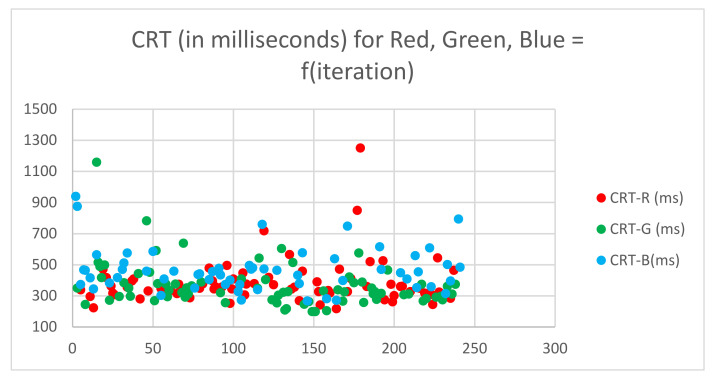
CRT (ms) for each color as function of iteration.

Two 5-min runs were made, and the results are plotted in the Figure 16. If the button was not pressed correctly the first time, then that measurement was not taken into consideration for statistics. 

Across all colors, the number of wrong presses was around 10%, with an average CRT time of 416 ms in the first run, and 16% with 385 ms average CRT, in the second run.

## 5. Conclusions

The proposed eHealth SoI smart solutions for ageing well is an incorporated solution oriented to support elders, suffering by/being at risk of age related NCDs and/or MCIs, in staying in their familiar surroundings for as long as possible, while still be safe and optimally cared for by the stakeholders (volunteers, family members and professional caregivers).

The proposed system offers the possibility to incorporate other biometric sensors, such as blood sugar and pressure, body positioning, etc., though not tested in the current study, except for the pulse rate, oxygen saturation and temperature together with a cognitive assessment sensor in terms of elders’ choice reaction time measurements.

The work was structured based on Systems Engineering Methodology, making use of commercial MySignals Hardware (HW) Development Platform—eHealth and Medical IoT Development Platform for Arduino. The elder’s biometric parameters are acquired via wearable sensors and transmitted to the envisaged stakeholder through a short- and long-range communication protocols (Wi-Fi and Bluetooth4) by using embedded C++ and web applications.

From a software point of view, the MySignals HW shield implementation was suboptimal, the authors spent some effort to generate documentation and reverse-engineer (for porting to another, more capable, baseboard) and assemble everything into a working product. High-level drivers had to be written on top of original low-level drivers:

- issDisplay: Provides font selection, font orientation, backlight on/off.
- issBitmap [52]: Provides picture storage in 2 bpp and retrieval, with speed-optimized drawing capabilities, which speculate pixel-after-pixel datastream towards the SPI connected display.
- issWi-Fi with its counterpart in 8266-modified driver, replaced the slow stock driver (at least 2 times improvement).
- issBLE: An improved BT-BLE device manager, which accelerates data access by at least 3 times.
- issBodyPosition/issTemperature/issSPO2/issBloodPressure/issBodyScale: OOP-approach as derived classes from issModule (proper class hierarchy).

In addition, the authors developed a test-framework (issMeasurements), which can be easily ported on any embedded platform, to be able to track hotspots and current consumption, and provided in the digital report all software required to do so.

With regards to PCB-production halt, the authors started a design and implementation of a PCB capable to at least replace the original MySignals HW shield.

As required in the co-design sessions with stakeholders, the herein eHealth system presented a mechanical case concept developed from scratch, electronic adaptations, and software implementations for a system based on composite commercial platforms (MySignals, Arduino) highlighting specific filtering/processing biometric signals, lowering range transmission rate and power consumption. Another point to be mentioned is the reason authors considered implementing CRT tests in embedded way, being that most studies they found were using a mouse connected to a computer with OS—and that could potentially add a large delay that might not even be deterministic. A cognitive assessment sensor via CRT has been added in order for the proposed eHealth system to provide specific NCD and cognitive separate and interconnected views via non-obtrusive biometric assessment means, with the potential to consolidate the future literature by exploiting the correlations between physiological and cognitive data and generating predictive analysis into the realm of eldercare. As planned in the SAVE AAL Project, when the physical access to elders will be available in the context of COVID-19 pandemics, the system demonstration of the pilots from Romania, Italy, and Hungary will gather the needed data to update and consolidate the relationships between NCDs and MCIs, with paramount potential in contributing to the relevant literature by using the proposed eHealth Internet-connected embedded system defined and implemented on elders’ needs as elicited during COVID-19 pandemics. 

## Figures and Tables

**Table 1:** ATMEGA2560 program memory occupied for each module in Bytes.

Software Module Name	Occupied Program Memory (in Bytes)	Occupied Program Memory (Old Implementation) (in Bytes)
Bitmap	19,968	Not available
issDisplay	26,648	9080
issWi-Fi5	1920	2134
issBattery	634	Not available
issTemperature	2198	2546
issBodyPositionSensor	1890	3782
issSpirometer	2022	3780
issSpO2	1278	774
issBodyScale	3588	2984
issBloodPressure	2346	6096
issGlucometer	1566	1018
Software glue logic	110	444
TOTAL	66,642	35,192

**Table 2:** Range of communication and consumed power per SoC.

Wireless Card/SoC	Technology	Range (m)	Typical Current (mA)	Peak Current (mA)	Comments
ESP8266	802.11 b/g/n	385	172	250	2400 MHz
XB24	ZigBee	110	33	40	2400 MHz
SX1272	LoRa	2000+	109	125	868 MHz
FS1000A	433-ASK	94	38	40	433 MHz

**Table 3:** Total current measurements extracted in several conditions of e-Health system operation.

Scenario	minIcc(mA)	avgIcc (mA)	maxIcc(mA)	Time Duration (us)
display with backlight	86	125	164	32
display without backlight	62	120	170	36
drawString	73	121	179	76
display init	73	153	170	6,567,216

**Table 4:** Improvements made in changing the AT-command stack of the ESP8266 802.11 chipset.

Operation	Min Required Time (ms)	Max Required Time (ms)	Avg Required Time (ms)
Old, default AT-command stack	15,075	18,091	15,100
New stack	7046	10,046	7273

**Table 5:** Old stack time-spending.

Communication Phase	Min (ms)	Max (ms)	Avg (ms)
CIPSTART	1009	4022	1028
CIPSEND	13,058	13,801	13,065
CIPCLOSE	1005	1008	1006

**Table 6:** Time measurements for various window-size of moving average, running on a 16 MHz ATMEGA 2560 MCU.

Filter Type	Window Size (Samples)	Total Size (Samples)	Total Measured Processing Time @ 16 MHz (in Microseconds). Resolution: 4 us	Approx. Window Processing Time (in Microseconds)
Moving Average	1	1000	15,232	15.23
	2	1000	16,252	16.26
	4	1000	18,324	18.39
	10	1000	24,504	24.75
	100	1000	117,228	130.25
	500	1000	541,776	1,083.55
	1000	1000	1,044,372	1,044,372.00

**Table 7:** Time measurements for various window-size of median filter, running on a 16 MHz ATMEGA 2560 MCU.

Filter Type	Window Size (Samples)	Total Size (Samples)	Total Measured Processing Time @ 16 MHz (in Microseconds). Resolution: 4 us	Approx. Window Processing Time (in Microseconds)
Median filtering (O(N^2^) sorting was used)	1	1000	3888	3.88
	2	1000	7504	7.51
	4	1000	18,524	18.59
	10	1000	82,124	82.95
	100	1000	5,978,944	6643.27

**Table 8:** Probability of Choice Reaction Time (CRT) stimulus occurrence.

Color	Probability of Occurrence (%)
Red	32.22
Green	34.73
Blue	33.05

## Data Availability

The data presented in this study are openly available at [53].
